# Experimental observation of dual magnetic states in topological insulators

**DOI:** 10.1126/sciadv.aav2088

**Published:** 2019-02-08

**Authors:** Wenqing Liu, Yongbing Xu, Liang He, Gerrit van der Laan, Rong Zhang, Kang Wang

**Affiliations:** 1 York-Nanjing Joint Center (YNJC) for Spintronics and Nanoengineering, School of Electronics Science and Engineering, Nanjing University, Nanjing 210093, China.; 2 Department of Electronic Engineering, Royal Holloway University of London, Egham TW20 0EX, UK.; 3 Department of Electronic Engineering, The University of York, York YO10 5DD, UK.; 4 Department of Electrical and Computer Engineering, Department of Materials Science and Engineering, and Department of Physics, University of California, Los Angeles, Los Angeles, CA 90095, USA.; 5 Magnetic Spectroscopy Group, Diamond Light Source, Didcot OX11 0DE, UK.

## Abstract

The recently discovered topological phase offers new possibilities for spintronics and condensed matter. Even insulating material exhibits conductivity at the edges of certain systems, giving rise to an anomalous quantum Hall effect and other coherent spin transport phenomena, in which heat dissipation is minimized, with potential uses for next-generation energy-efficient electronics. While the metallic surface states of topological insulators (TIs) have been extensively studied, direct comparison of the surface and bulk magnetic properties of TIs has been little explored. We report unambiguous evidence for distinctly enhanced surface magnetism in a prototype magnetic TI, Cr-doped Bi_2_Se_3_. Using synchrotron-based x-ray techniques, we demonstrate a “three-step transition” model, with a temperature window of ~15 K, where the TI surface is magnetically ordered while the bulk is not. Understanding the dual magnetization process has strong implications for defining a physical model of magnetic TIs and lays the foundation for applications to information technology.

## Teaser

The dual magnetic states in topological insulators reveal the interplay between two ordering phenomena: magnetism and topology.

## INTRODUCTION

**Fig. 1:**
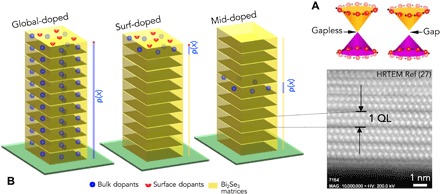
Experimental configuration. (**A**) Conceptual illustration of the Dirac fermion states of the Bi_2_Se_3_ topological insulator. In the magnetically doped Bi_2_Se_3_ (right), a bandgap is present between the upper and lower Dirac cones. (**B**) Sample configuration of the global-, surf-, and mid-doped Bi_2_Se_3_. Each cuboid represents one Bi_2_Se_3_ quintuple layer (QL).

Three-dimensional (3D) topological insulators (TIs) feature novel phases of quantum matter with sharp transitions in the electronic structure near their surfaces. Unlike the divergent electronic properties of surface and bulk regions of all solids owing to the inevitable termination of the periodic lattice structure when approaching the boundaries, TIs present a new class of nontrivial surface states arising from intrinsic strong spin-orbit coupling and characterized by Rashba spin texture (*1*–*5*). These low-dimensional surface states are immune to localization caused by disorders as long as the disorder potential is time-reversal invariant and therefore have strong implications for emerging technologies such as dissipationless transport and quantum computation (*6*, *7*). Breaking the time-reversal invariance by introducing magnetic perturbation, on the other hand, reveals a complex phenomenology associated with a tunable excitation bandgap of the surface spectrum (*8*, *9*), as illustrated in Fig. 1A. Such a magnetic TI system resembles that of a massive Dirac fermion, which represents an ideal laboratory to study the interplay between magnetism and topology (*10*–*15*).

While the presence of the metallic surface state has been well studied (*1*–*14*), experimental evidence on the magnetic properties of the TI surface is far from conclusive. It has been proposed that, in magnetic TIs, ferromagnetism can be developed not only through the van Vleck mechanism (*10*, *16*), by which magnetic ions are directly coupled through the local valance electrons, but also the carrier-mediated Ruderman-Kittel-Kasuya-Yosida (RKKY) interaction when Fermi energy (*E*_F_) is close to the Dirac point (*17*, *18*). The surface versus bulk magnetization of a magnetic TI reflects a central question of topology: the role of dimensionality. In general, ordered phenomena, e.g., magnetic, superconducting order, and lattice stability etc., show fragility when the dimension of the system decreases (*T*_c_^3D^ > *T*_c_^2D^) (here, *T*_c_ refers to the critical temperature of ordered phenomena in general). However, in the case of 3D TIs, the opposite can occur, such that *T*_c_^surface^ > *T*_c_^bulk^, because of the unusual properties of the mediated helical electrons (*19*). Measurements show that the Dirac gap of a TI emerges independently of the bulk magnetic ordering (*9*). A number of recent reports on 3D magnetically-doped TIs including Mn-doped Bi_2_(TeSe)_3_ (*20*), Cr-doped (BiSb)_2_Te_3_ (*21*), Cr-doped (Bi*_x_*Sb_1−*x*_)_2_Te_3_ (*16*), and V-doped Sb_2_Te_3_ (*22*, *23*) seem to be arriving at a consensus that surface magnetization is primarily predominated by the RKKY mechanism, while that of the bulk is steered by the van Vleck interaction. Nevertheless, the key issue of the dual magnetic states of 3D TIs, i.e., that the surface has a different magnetic moment and ordering temperature than that of the bulk, has yet to be established. Delicate techniques such as time-resolved angular-resolved photoemission spectroscopy, have been performed to distinguish bulk and surface electron-phonon coupling of the bare TI (*24*). With respect to magnetic TIs, β-nuclear magnetic resonance was used for depth profiling the electronic wave functions at topological surfaces (*25*).

It is possible to distinguish the surface moment of a magnetic TI from that of the bulk using the synchrotron-based x-ray absorption technique. This method is based on the modified surface band structure, i.e., surface-atom core-level shift (*26*–*28*), which is reflected in a different surface valance state of metallic elements and can be experimentally observed in their characteristic x-ray absorption spectroscopy (XAS) spectra. Tuning the absorption to the magnetic resonant edges, x-ray magnetic circular dichroism (XMCD) can be obtained for the surface and bulk dopants, respectively, revealing the magnetic ground state and temperature dependence in an unambiguous surface bulk–resolved manner. Here, we present a study of a prototype magnetic TI, i.e., Cr-doped Bi_2_Se_3_ in its ultrathin limit that is expected to give rise to the quantum anomalous Hall effect (*10*). Three kinds of samples were measured as illustrated in Fig. 1B. In the global-doped Bi_2_Se_3_ thin films, the Cr dopants are uniformly distributed throughout the sample, whereas in the modulation-doped Bi_2_Se_3_ thin films, the dopants are only introduced either into the topmost (referred as surf-doped) or the middle (referred as mid-doped) quintuple layer (QL), respectively. This was achieved by accurately controlling the dopant distribution profiles along the growth direction using the slow-deposition molecular beam epitaxy technique (see the Supplementary Materials).

## RESULTS

**Fig. 2:**
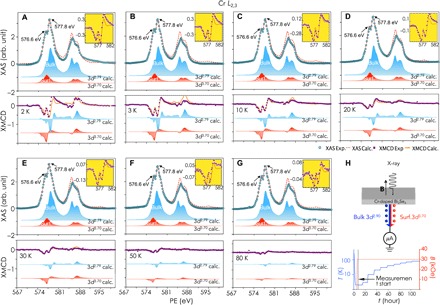
The global-doped Bi_2_Se_3_. (**A** to **G**) Typical total XAS and XMCD and their deconvoluted spectra of the global-doped Bi_2_Se_3_ at 2 to 80 K, respectively. The (percentage) XMCD intensity at the Cr *L*_3_ edge decreases with the increasing temperature within the measured range. The best fitting was obtained by a linear superposition of *d*_surf_^3.70^ and *d*_bulk_^2.79^, with ~1:3 for the total XAS and ~1:2 for the XMCD. (**H**) Schematic diagram of the experimental set up and the measurement processes. Circularly polarized x-rays were used in normal incidence with respect to the sample plane and parallel to the applied magnetic field. Samples were cooled down to 2 K without magnetic field, and data were collected in the warm-up cycle.

We first address the dual magnetic states observed in the global-doped sample, i.e., 10-nm 3% Cr-doped Bi_2_Se_3_ epitaxial thin films. Figure 2 (A to G) present the measured total XAS and the XMCD spectra at the Cr *L*_2,3_ edge of the global-doped Bi_2_Se_3_ thin film obtained in the total electron yield (TEY) mode (see the Supplementary Materials). The total XAS of Cr shows remarkable multiplet structures separated by 1.2 eV at both spin-orbit split core levels, suggesting a mixture of divalent and trivalent Cr. Atomic multiplet calculations were performed to simulate the electric-dipole transitions, i.e., 3*d^n^* → 2*p*^5^3*d*^*n* + 1^, to deconvolute the hybridized spectra (see the Supplementary Materials) (*29*, *30*). The best fit was obtained by a linear superposition of covalent Cr *d*^3.70^ (divalent) and *d*^2.79^ (trivalent) with ~1:3 for the total XAS, while this ratio is ~1:3 at 3 K and goes all the way up to ~1:1.5 at 80 K for the XMCD spectra. This suggests that, compared to the Cr *d*^3.70^, the Cr *d*^2.79^ loses magnetic ordering significantly faster with increasing temperature. The branching ratio (*31*), which quantifies the relative intensity of the *L*_3_ edge in the total *L*_2,3_ XAS intensity of the hybrid Cr *L*_2,3_ XAS spectra, is 0.63, standing in between 0.61 for *d*^2.79^ and 0.68 for *d*^3.70^ for the octahedral crystal-field symmetry.

Qualitatively, the contribution of the surface can be identified by comparing the spectra obtained in the bulk-sensitive total fluorescence yield (TFY) detection with that in the surface-sensitive TEY detection. While TEY at normal incidence probes only the top ~5 nm near the surface, TFY has a penetration depth of more than 100 nm (*31*, *32*). In the global-doped Bi_2_Se_3_ thin film, the low-energy peak (*d*^ 3.70^) is notably absent in the TFY spectrum, indicating that this peak originates from the top few atomic layers of the sample. First-principles density functional theory simulations confirm that this *d*^3.70^ state is unlikely coming from any form of defects within Cr-doped Bi_2_Se_3_ (*33*). Note that the TEY intensity is attenuated by an exponentially decaying electron-escape probability. Therefore, in the total TEY-XAS spectra, the ratio of the Cr *d*^3.70^ to Cr *d*^2.79^ shows ~1:3 other than an unweighted sum of ~1:9 that one may tentatively assume.

**Fig. 3:**
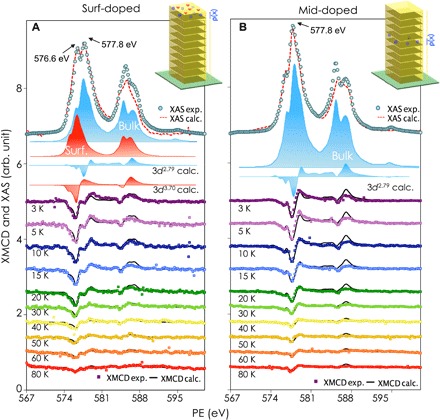
The modulation-doped Bi_2_Se_3_. Typical total XAS and XMCD and their deconvoluted spectra of the (**A**) surf-doped and (**B**) mid-doped Bi_2_Se_3_ at 3 to 80 K, respectively. For the surf-doped Bi_2_Se_3_, the best fitting was obtained by a linear superposition of *d*_surf_^3.70^ and *d*_bulk_^2.79^, with ~4:5 for the total XAS and ~2:1 for the XMCD. No appreciable Cr *d*_surf_^3.70^ but only Cr *d*_bulk_^2.79^ was obtained from the mid-doped Bi_2_Se_3_.

We now have a picture that the two deconvoluted Cr *d*^3.70^ and Cr *d*^2.79^ spectra uniquely represent the surface and the bulk properties of the Bi_2−*x*_Cr*_x_*Se_3_ and denote them as *d*_surf_^3.70^ and *d*_bulk_^2.79^, respectively, hereafter. Figure 3 (A and B, respectively) presents the total XAS and XMCD spectra of the modulation-doped Bi_2_Se_3_ thin films with an effective doping of 1.2% from 3 to 80 K. The deconvolution of the total XAS gives the ratio of ~4:5 for the contribution of the Cr *d*_surf_^3.70^ and the Cr *d*_bulk_^2.79^ for the surf-doped sample, whereas no appreciable Cr *d*_surf_^3.70^ but only Cr *d*_bulk_^2.79^ was observed from the mid-doped sample. Note that even the surf-doped Bi_2_Se_3_ contains both Cr *d*_surf_^3.70^ and Cr *d*_bulk_^2.79^ with the later arising from the lower atomic sublayers of the first QL (see Fig. 1B). This is consistent with that observed for the global-doped sample.

**Fig. 4:**
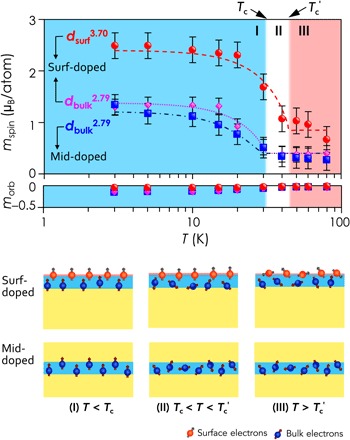
*M*-*T* relationships. (**Top**)The XAS/XMCD-derived *m*_spin_ and *m*_orb_ of the *d_surf_*^3.70^ and *d_bulk_*^2.79^ versus temperature (*T*) at 3 to 80 K of the modulation-doped Bi_2_Se_3_ thin films. The dashed lines are the best fit within the mean-field approximation. (**Bottom**) Schematic illustration of the three-step transition: Both the surface and bulk are magnetically ordered below *T*_c_ (phase I); between *T*_c_ and *T*_c_′, the surface retains magnetization while the bulk does not anymore (phase II); eventually, beyond *T*_c_′, both the surface and bulk lose their magnetic orders (phase III).

Figure 4 presents the XMCD-derived spin (*m*_spin_) and orbital (*m*_orb_) magnetic moments versus temperature of the global- and the modulation-doped Bi_2_Se_3_, respectively, by applying sum rules to the separate XAS and XMCD spectra (see the Supplementary Materials). The XMCD-derived *m*_spin_ and *m*_orb_ of both the Cr *d*_surf_^3.70^ and Cr *d*_bulk_^2.79^ have opposite signs, corresponding to antiparallel alignment of the spin and orbital magnetization. This agrees with the Hund’s rule for Cr, whose 3*d* shell is less than half full. For the global-doped Bi_2_Se_3_, we obtained a remarkable *m*_spin_ = (3.44 ± 0.30) μ_B_/atom and a small negative *m*_orb_ = (−0.06 ± 0.03) μ_B_/atom for the Cr *d*_surf_^3.70^, while those for the Cr *d*_bulk_^2.79^ are *m*_spin_ = (1.65 ± 0.30) μ_B_/atom and *m*_orb_ = (−0.09 ± 0.03) μ_B_/Cr at 3 K. For the modulation-doped Bi_2_Se_3_, the magnetization is slightly suppressed because of the reduced thickness of the doped region. We obtained *m*_spin_ = (2.49 ± 0.25) μ_B_/atom and *m*_orb_ = (−0.04 ± 0.02) μ_B_/atom for the Cr *d*_surf_^3.70^ of the surf-doped Bi_2_Se_3_. For the Cr *d*_bulk_^2.79^, the magnetic moments extracted from the surf- and the mid-doped Bi_2_Se_3_ are identical within the experimental accuracy, namely, *m*_spin_ = (1.30 ± 0.10) μ_B_/atom and *m*_orb_ = (−0.14 ± 0.02) μ_B_/atom for the former and *m*_spin_ = (1.34 ± 0.10) μ_B_/atom and *m*_orb_ = (−0.15 ± 0.02) μ_B_/atom for the latter, respectively. While *m*_orb_ of the Cr *d*_bulk_^2.79^ for all the three samples is notably large, that of the Cr *d*_surf_^3.70^ is nearly quenched, which may be attributed to a slight distortion of the lattice symmetry of the surface.

## DISCUSSION

In line with the electrical magnetotransport measurements (*33*), the XMCD-derived *m*_spin_ exhibits a Curie-like behavior, pointing to a ferromagnetic phase of the Bi_2−*x*_Cr*_x_*Se_3_ thin film at low temperatures. The fact that the *m*_spin_ of the Cr *d*_surf_^3.70^ and the Cr *d*_bulk_^2.79^ of the global-doped Bi_2_Se_3_ show distinct temperature dependences points to the presence of dual magnetic states processing within one sample. As shown in Fig. 4, the Cr *d*_surf_^3.70^ exhibits more robust magnetization in both the magnitude of moment and the ordering temperature in comparison to that of the Cr *d*_bulk_^2.79^. Fitting the temperature-dependent magnetization within the mean-field approximation, i.e., *M*(*T*) ∝ (1 − *T*/*T*_c_)^γ^, where γ represents the critical exponent, we obtained *T*_c_ = (31.0 ± 3.2) K and (31.3 ± 2.9) K for the Cr *d*_bulk_^2.79^ in the surf- and the mid-doped Bi_2−*x*_Cr*_x_*Se_3_, respectively, and *T*_c_′ = (46.4 ± 2.5) K for the Cr *d*_surf_^3.70^ in the surf-doped Bi_2−*x*_Cr*_x_*Se_3_. Table 1 summarizes the XMCD-derived *m*_s_, *T*_c_, and γ of the modulation-doped Bi_2_Se_3_ thin films.

Keeping in mind that the Cr *d*_bulk_^2.79^ and Cr *d*_surf_^3.70^ correspond to the two respective magnetization modes of the bulk and the surface, we conclude a “three-step-transition” model for the magnetic TIs against temperature. As illustrated in the upper row of Fig. 4: During phase I, both the surface and bulk are magnetically ordered below *T*_c_; between *T*_c_ and *T*_c_′ (phase II), the surface retains magnetization while the bulk does not any longer; eventually, above *T*_c_′ (phase III), both the surface and the bulk lose their magnetic ordering. Note that, because electrical measurements are sensitive to the bulk, the transport-derived magnetically ordered temperatures (*33*) have a different physical meaning as those obtained using dichroic spectra and are rather close to the *T*_c_ of the Cr *d*_bulk_^2.79^. It is known that, in diluted magnetic semiconductors, ferromagnetic ordering is set via carrier-mediated exchange, which depends on the carrier concentration and, in turn, on the magnetic dopant concentration (*34*). The high density of free carriers required in these systems, however, is unsuitable for TIs (*33*). Theoretical predictions (*17*) indicate that the surface state–mediated spin-spin interaction is naturally ferromagnetic and even the bulk TI remains paramagnetic; experiments confirm that, in the magnetically doped Bi_2_Se_3_ systems, the Dirac gap in the surface spectrum can be present without bulk magnetic ordering (*8*, *9*). A sharp transition of the magnetic susceptibility at the surface of TIs has been predicted (*35*) and demonstrated in experiment (*25*). Calculation (*19*) of the surface magnetic ordering of TIs has estimated values of 17.5 and 29 K, depending on the lattice model selected, for this temperature “window.” This estimate compares well with our observation.

**Table 1:** Summary of the XMCD-derived *m*_s_, *T*_c_, and γ of the modulation-doped Bi_2_Se_3_ thin films.

**Sample**	**Cr dopants**	***m*_s_ (μ_B_/atom)**	***T*_c_ (K)**	**γ**
Surf-doped	*d*_surf_^3.70^	2.49 ± 0.25	46. 4 ± 2.5	0.40 ± 0.14
	*d*_bulk_^2.79^	1.30 ± 0.10	31.0 ± 3.2	0.29 ± 0.17
Mid-doped	*d*_bulk_^2.79^	1.34 ± 0.10	31.3 ± 2.9	0.58 ± 0.10

To conclude, we have defined and validated an experiential approach to determine the magnetic ground state in a “surface-specific” manner using synchrotron-based x-ray techniques. We have unambiguously observed an enhanced surface magnetic ordering of the Bi_2−*x*_Cr*_x_*Se_3_ systems with a significantly large surface magnetic moment and high ordering temperature. We have demonstrated a three-step-transition model, in which a temperature window of ~15 K exists where the surface of the TI is magnetically ordered but the bulk is not. Future work to explore the tuning of this window and understand the dual magnetization process will have strong relevance to refining the physical model of magnetic TIs and lays the foundation for applications to emerging spintronic technologies.

## MATERIALS AND METHODS

XAS and XMCD measurements at the Cr *L*_2,3_ absorption edges of the Bi_2−*x*_Cr*_x_*Se_3_/Si(111) thin film were performed on beamline I10 at Diamond Light Source, UK. Circularly polarized x-rays with ~100% polarization were used in normal incidence with respect to the sample plane and parallel to the applied magnetic field, as illustrated in Fig. 2H. The XMCD was obtained by taking the difference of the XAS spectra, i.e., σ^+^ − σ^−^, by flipping the x-ray helicity at a fixed magnetic field of 30 kOe. The total XAS, on the other hand, was obtained by averaging over the two polarizations, i.e., (σ^+^ *+* σ^−^)/2. The intensity and the detailed line shape of the total XAS spectra reveal information of the Cr impurities in different valance states, while those of the XMCD spectra indicate the corresponding magnetic ground states. Atomic multiplet theory was used to calculate the electric-dipole transitions 3*d^n^* → 2*p*^5^3*d*^*n* + 1^, where the spin-orbit and electrostatic interactions were treated on an equal footing (*36*). The wave functions of the initial- and final-state configurations were calculated in intermediate coupling using the Cowan’s atomic Hartree-Fock (HF) code with relativistic corrections. The atomic electrostatic interactions include the 2*p*-3*d* and 3*d*-3*d* Coulomb and exchange interactions, which are reduced to 70% of their atomic HF value to account for the intra-atomic screening (*36*). Hybridization effects were included by mixing 3*d^n^* with 3*d*^*n* + 1^*L* configurations, where *L* represents a hole on the neighboring atoms in states of appropriate symmetry. The Cr *L*_3_ (*L*_2_) line spectra were broadened by a Lorentzian with a half width at half maximum of Γ = 0.3 eV (0.4 eV) for intrinsic lifetime broadening and a Gaussian with an SD of σ = 0.15 eV for instrumental broadening.

## Supplementary Material

http://advances.sciencemag.org/cgi/content/full/5/2/eaav2088/DC1

## SUPPLEMENTARY MATERIALS

Supplementary material for this article is available at http://advances.sciencemag.org/cgi/content/full/5/2/eaav2088/DC1 Section S1. Sample preparation Section S2. XAS/XMCD measurement Section S3. Multiplet calculations Section S4. Sum-rules analysis Fig. S1. Schematic diagram of the experimental setup for XAS and XMCD measurement. Fig. S2. Deconvolution of the mixed Cr valences. Fig. S3. The sum-rules analysis. Table S1. Summary of the XMCD-derived *m*_spin_ for the global-, surf-, and mid-doped Cr-doped Bi_2_Se_3_, respectively, at 3 K. References (*37*–*43*)
