# Supplementary material for: Experimental observation of dual magnetic states in topological insulators
Source: Sci Adv. 2019 Feb 8;5(2):eaav2088. doi: 10.1126/sciadv.aav2088 (PMC6368422; doi:10.1126/sciadv.aav2088)
Supplement: http://advances.sciencemag.org/cgi/content/full/5/2/eaav2088/DC1 [file supp_5_2_eaav2088__index.html]

Science Advances | Science Advances

## Supplementary Materials

**This PDF file includes:**

- Section S1. Sample preparation
- Section S2. XAS/XMCD measurement
- Section S3. Multiplet calculations
- Section S4. Sum-rules analysis
- Fig. S1. Schematic diagram of the experimental setup for XAS and XMCD measurement.
- Fig. S2. Deconvolution of the mixed Cr valences.
- Fig. S3. The sum-rules analysis.
- Table S1. Summary of the XMCD-derived *m*spin for the global-, surf-, and mid-doped Cr-doped Bi2Se3, respectively, at 3 K.
- References (*37*–*43*)

Download PDF

**Files in this Data Supplement:**

- Adobe PDF - aav2088\_SM.pdf
