# Supplementary material for: Experimental observation of dual magnetic states in topological insulators
Source: Sci Adv. 2019 Feb 8;5(2):eaav2088. doi: 10.1126/sciadv.aav2088 (PMC6368422; doi:10.1126/sciadv.aav2088)
Supplement: http://advances.sciencemag.org/cgi/content/full/5/2/eaav2088/DC1 [file aav2088_SM.pdf]

Science Advances
AAAS

advances.sciencemag.org/cgi/content/full/5/2/eaav2088/DC1

Supplementary Materials for

Experimental observation of dual magnetic states in topological insulators

Wenqing Liu, Yongbing Xu*, Liang He*, Gerrit van der Laan, Rong Zhang, Kang Wang*

*Corresponding author. Email: yongbing.xu@york.ac.uk (Y.X.); heliang@nju.edu.cn (L.H.); wang@seas.ucla.edu (K.W.)

Published 8 February 2019, *Sci. Adv.* **5**, eaav2088 (2019)
DOI: 10.1126/sciadv.aav2088

This PDF file includes:

Section S1. Sample preparation
Section S2. XAS/XMCD measurement
Section S3. Multiplet calculations
Section S4. Sum-rules analysis
Fig. S1. Schematic diagram of the experimental setup for XAS and XMCD measurement.
Fig. S2. Deconvolution of the mixed Cr valences.
Fig. S3. The sum-rules analysis.
Table S1. Summary of the XMCD-derived m_{spin} for the global-, surf-, and mid-doped Cr-doped Bi_2Se_3, respectively, at 3 K.
References (*37–43*)

Section S1. Sample preparation

The 10 nm 3% Cr-doped Bi_2Se_3 or $Bi_{1.94}Cr_{0.06}Se_3$ thin films used in this study were grown in an ultra-high vacuum (UHV) chamber using a Perkin-Elmer molecular beam epitaxy (MBE) system on Si(111) substrates using the well-established recipe (*37*, *38*, *39*). High-resistivity Si(111) substrates are first cleaned with standard RCA cleaning method. The substrates are then treated by hydrofluoric acid wet etching so that the surface dangling bonds are saturated by a layer of hydrogen atoms and the (111) surface is 1 x 1 reconstructed. The substrates were kept at 200°C in UHV until the two-dimensional streak pattern appeared as monitored by the real-time reflection high-energy electron diffraction (RHEED). High-purity Bi (99.9999%) was evaporated from a conventional effusion cell at 470°C, while Se (99.99%) was created from a cracker cell (SVTA) at 270°C. The desired doping concentrations of Cr was obtained by evaporating Cr (99.99%) from the effusion cell at different temperatures and then calibrated by energy dispersive X-ray analysis. Immediately after the growth of the $Bi_{1.94}Cr_{0.06}Se_3$ thin films, 2 nm Al was *in-situ* evaporated onto the sample to protect it from oxidation and environmental doping during the transport to the synchrotron facility.

All the global- and the modulation-doped Bi_2Se_3 thin films used in this study have identical thicknesses (10 nm). In the global-doped samples, the Cr dopants are uniformally distributed throughout the sample, wheares in the modulation-doped Bi_2Se_3, the dopants were only introduced into the topmost (referred to as *surf-doped*) or in the middle (referred to as *mid-doped*) one quintuple layer (QL), respectively, as described in the main text. This was achieved by the slow deposition rate (i.e., ~1 QL per minute as calibrated from the RHEED oscillation patterns) and the layer-by-layer growth geometry of Bi_2Se_3 matrix.[40, 41, 42] It allows us to accurately control the Cr-dopant distribution profiles along the epitaxial growth direction (deviation < ±0.1 nm) (*40*). Note that the ‘surface doping’ in this work has a different physical meaning as in ref. (*41*), where metallic adatoms are deposited onto the surface of a bare TI. Here we discuss the as-grown Bi_2Se_3 with the topmost 1 QL doped with Cr ions, in order to make it comparable with the mid-doped Bi_2Se_3 (both contain only 1 QL of magnetic impurities). The merit of this design is to eliminate any influence of the thickness of the magnetically doped layer.

Section S2. XAS/XMCD measurement

XAS and XMCD measurements at the Cr $L_{2,3}$ absorption edges of the $Bi_{2-x}Cr_xSe_3$/Si(111) thin film were performed on beamline I10 at Diamond Light Source, UK. Circularly polarized X-rays with ~100% polarization were used in normal incidence with respect to the sample plane and parallel to the applied magnetic field, as illustrated in fig. S1. The XMCD was obtained by taking the difference of the XAS spectra, i.e., $\sigma^+ - \sigma^-$, by flipping the X-ray helicity at a fixed magnetic field of 30 kOe (*38*, *39*). The total XAS, on the other hand, was obtained by averaging over the two polarizations, i.e., $(\sigma^+ + \sigma^-)/2$. The intensity and the detailed line shape of the total XAS spectra reveal information of the Cr impurities in different valance states; whilst those of the XMCD spectra indicate the corresponding magnetic ground states.

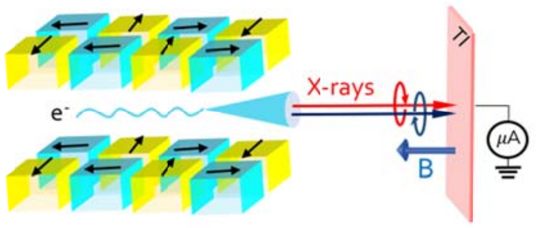

Fig. S1. Schematic diagram of the experimental setup for XAS and XMCD measurement.

Section S3. Multiplet calculations

Atomic multiplet theory is used to calculate the electric-dipole transitions $3d^n \rightarrow 2p^5 3d^{n+1}$, where the spin-orbit and electrostatic interactions are treated on an equal footing.[33] The wave functions of the initial- and final-state configurations are calculated in intermediate coupling using Cowan's atomic Hartree-Fock (HF) code with relativistic corrections (*36*). The atomic electrostatic interactions include the $2p$-$3d$ and $3d$-$3d$ Coulomb and exchange interactions, which are reduced to 70% of their atomic HF value to account for the intra-atomic screening (*36*). Hybridization effects are included by mixing $3d^n$ with $3d^{n+1}\underline{L}$ configurations, where $\underline{L}$ represents a hole on the neighboring atoms in states of appropriate symmetry. The Cr L_3 (L_2) line spectra are broadened by a Lorentzian with a half-width at half-maximum of Γ = 0.3 eV (0.4 eV) for intrinsic lifetime broadening and a Gaussian with a standard deviation of σ = 0.15 eV for instrumental broadening.

As shown in fig. S2, the best agreement between the experiment and the calculation of the total XAS was obtained by a linear superposition of $3d^{3.70}$ and $3d^{2.79}$ with ~1:3 for the global-doped Bi_2Se_3 and ~4:5 for

the surf-doped Bi_2Se_3, respectively. No appreciable Cr $d_{surf}^{3.70}$ but only Cr $d_{bulk}^{2.79}$ was obtained from the mid-doped Bi_2Se_3. These are consistent with the global-, mid-, and surf-doped sample configuration given that the TEY intensity is attenuated by an exponentially decaying electron-escape probability. We therefore obtained the picture that the deconvoluted Cr $d^{3.70}$ and Cr $d^{2.79}$ spectra uniquely represent the surface and the bulk properties of the $Bi_{2-x}Cr_xSe_3$. They are denoted as $d_{surf}^{3.70}$ and $d_{bulk}^{2.79}$, respectively, in the paper.

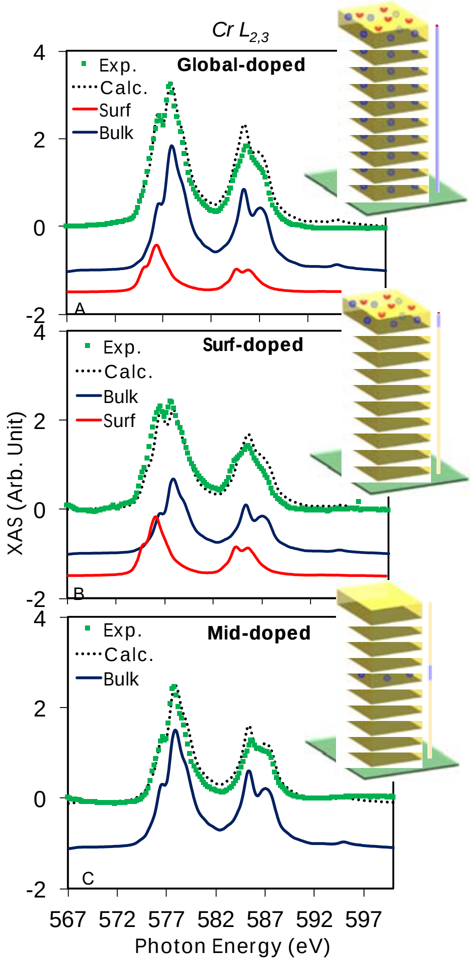

Fig. S2. Deconvolution of the mixed Cr valences. Typical total XAS spectra of the (**A**) global-, (**B**) surf-, and (**C**) mid-doped Bi_2Se_3. Inset: sample configuration of the global-, surf-, and mid-doped Bi_2Se_3, used in this study.

Section S4. Sum-rules analysis

The spin (m_{spin}) and orbital (m_{orb}) magnetic moments of the Cr-doped Bi_2Se_3 epitaxial thin films were calculated by applying the sum rules (*42*, *43*) to the integrated XMCD and summed XAS spectra of the Cr $L_{2,3}$ edges, based on

$$m_{orb} = -\frac{4}{3} n_d \frac{\int_{L_{2,3}} (\sigma^+ - \sigma^-)\, dE}{\int_{L_{2,3}} (\sigma^+ + \sigma^-)\, dE}$$

$$m_{spin} + < T_z > = -n_d \frac{6\int_{L_3} (\sigma^+ - \sigma^-)\, dE - 4\int_{L_{2,3}} (\sigma^+ - \sigma^-)\, dE}{\int_{L_{2,3}} (\sigma^+ + \sigma^-)\, dE} SC$$

Eq. S1

where *E*, n_h, SC, and $<T_z>$ represent the photon energy, the number of *d* holes, the spin correction (SC) factor, and the magnetic dipole term, respectively. In order to exclude the non-magnetic contribution of the XAS spectra an arctangent-based step function is used to fit the threshold. The spectral overlap or *j-j* mixing[33] had to be taken into account because of the relatively small spin-orbit coupling in the Cr 2*p* level. The value of SC, i.e. 2.0 ± 0.2 for Cr, was estimated by calculating the $L_{2,3}$ multiplet structure for a given ground state, applying the sum rule on the calculated XMCD spectrum, and comparing the result with the spin moment calculated directly for this ground state.[39] Furthermore, m_{spin} needs to be corrected for the magnetic dipole term $<T_z>$, however, its contribution is small for a Cr t_{2g}^3 configuration, giving an error < 5%. Using n_h = 6.30 for the Cr $d^{3.70}$ and 7.21 for the Cr $d^{2.79}$ based on the fitting with the multiplet calculations, we obtained a remarkable m_{spin} = (3.44 ± 0.30) μ_B/atom and a small negative m_{orb} = (-0.06 ± 0.03) μ_B/atom for the Cr $d_{surf}^{3.70}$ whilst those for the Cr $d_{bulk}^{2.79}$ are m_{spin} = (1.65 ± 0.30) μ_B/atom and m_{orb} = (-0.09 ± 0.03) μ_B/Cr at 3 K. For the modulation-doped Bi_2Se_3, the magnetization is slightly supressed because of the reduced thickness of the doped region. We obtained m_{spin} = (2.49 ± 0.25) μ_B/atom and m_{orb} = (-0.04 ± 0.02) μ_B/atom for the Cr $d_{surf}^{3.70}$ of the surf-doped Bi_2Se_3. For the Cr $d_{bulk}^{2.79}$, the magnetic moments extracted from the surf- and the mid-doped Bi_2Se_3 are identical within the experimental accuracy, namely m_{spin} = (1.30 ± 0.10) μ_B/atom and m_{orb} = (-0.14 ± 0.02) μ_B/atom for the former and m_{spin} = (1.34 ± 0.10) μ_B/atom and m_{orb} = (-0.15 ± 0.02) μ_B/atom for the latter, respectively. The XMCD-derived m_{spin} of the surface and the bulk of the three kinds of samples are summarized in table S1.

Table S1. Summary of the XMCD-derived m_{spin} for the global-, surf-, and mid-doped Cr-doped Bi_2Se_3, respectively, at 3 K.

Sample Configuration	Cr $d_{surf}^{3.70}$ (μ_B/atom)	Cr $d_{bulk}^{2.79}$ (μ_B/atom)
Global-doped	3.44 ± 0.30	1.65 ± 0.30
Surf-doped	2.49 ± 0.25	1.30 ± 0.10
Mid-doped	-	1.34 ± 0.10

The XMCD-derived m_{orb} and m_{spin} for both Cr $d_{surf}^{3.70}$ and $d_{bulk}^{2.79}$ have opposite signs, corresponding to an antiparallel alignment of the spin and orbital moment in Cr. This agrees with the Hund's rule for Cr, whose 3*d* shell is less-than-half full. The octahedral crystal-field interaction quenches m_{orb}, since the 3*d* electrons occupy the threefold degenerate majority-spin t_{2g} orbitals, leading to a nearly vanishing m_{orb}. In reality the octahedral symmetry is trigonally distorted to C_{3v} with three anions above and below the Cr ion, which can alter the m_{orb} but hardly affects the m_{spin} (*29*). Figure S3 presents typical pairs of XAS for opposite circular polarizations and the corresponding XMCD of the global-, surf-, and mid-doped Bi_2Se_3, respectively, obtained at 3 K. Noting that the integrated total XAS and XMCD spectra well saturate at ~ 590 eV, the selected integration range, i.e. up to 600 eV, is sufficient.

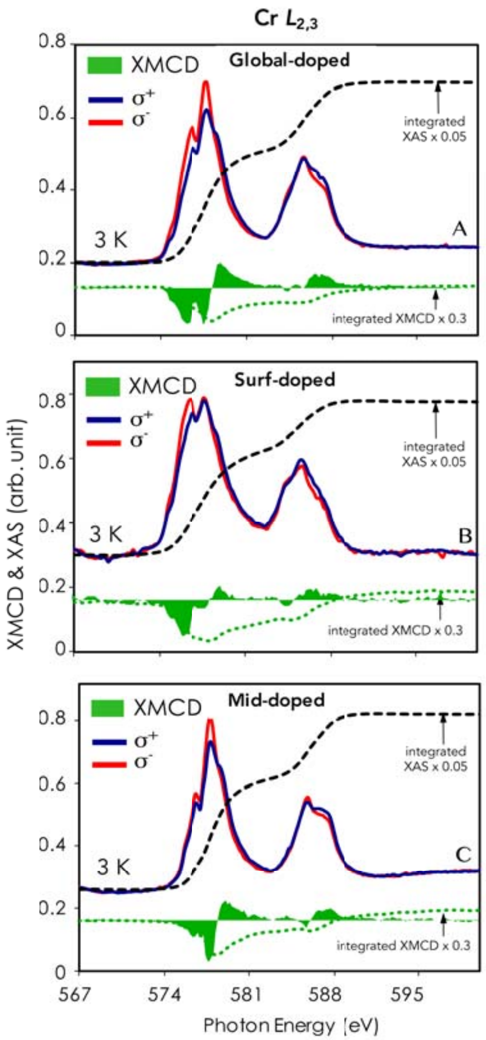

Fig. S3. The sum-rules analysis. Typical pair of XAS for opposite circular polarizations and corresponding XMCD of the (**A**) global- and (**B**) surf-, and (**C**) mid-doped Cr-doped Bi_2Se_3 obtained at 3 K. The dashed lines indicate the integrated total XAS and XMCD spectra averaged over the two Cr compositions. Data are offset and scaled for clarity.
